# The Nephrotoxicity of Drugs Used in Causal Oncological Therapies

**DOI:** 10.3390/curroncol29120760

**Published:** 2022-12-08

**Authors:** Janusz Hałka, Sebastian Spaleniak, Grzegorz Kade, Stefan Antosiewicz, Dawid Sigorski

**Affiliations:** 1 Department of Clinical Hematology, Warmian-Masurian Cancer Center of the Ministry of the Interior and Administration’s Hospital, Wojska Polskiego 37, 10-228 Olsztyn, Poland; 2 Department of Internal Diseases and Nephrodiabetology, Medical University of Lodz, Żeromskiego 113, 90-549 Lodz, Poland; 3 Warmian-Masurian Cancer Center of the Ministry of the Interior and Administration’s Hospital, Wojska Polskiego 37, 10-228 Olsztyn, Poland; 4 Military Institute of Aviation Medicine, Center of Aeromedical Examination and Occupational Medicine, Zygmunta Krasińskiego 54/56, 01-755 Warsaw, Poland; 5 Department of Oncology, Collegium Medicum, University of Warmia and Mazury, Aleja Warszawska 30, 11-082 Olsztyn, Poland; 6 Department of Oncology and Immuno-Oncology, Warmian-Masurian Cancer Center of the Ministry of the Interior and Administration’s Hospital, Wojska Polskiego 37, 10-228 Olsztyn, Poland

**Keywords:** nephrotoxicity, renal failure, acute kidney injury, chemotherapy, immunotherapy

## Abstract

In recent years, a dynamic development of oncology has been observed, resulting from the increasingly frequent occurrence of neoplasms and therefore, increasing population of patients. The most effective form of therapy for cancer patients is complex multidisciplinary specialized disease management, including nephro-oncology care. Different forms of renal function impairment are frequently diagnosed in cancer patients. They are caused by different co-morbidities existing before starting the oncologic treatment as well as the direct undesirable effects of this therapy which may cause temporary or irreversible damage of the urinary system—especially kidneys. According to different therapeutic programs, in such cases the degree of renal damage is often crucial for the possibility of further anti-cancer treatment. Medical personnel responsible for delivering care to oncology patients should be properly educated on current methods of prevention and treatment of renal complications resulting from anti-cancer therapy. The development of oncologic medicines design, including especially immuno-oncological agents, obliges us to learn new patomechanisms determining potential adverse effects, including renal complications. This publication is focused on the most important undesirable nephrotoxic effects of the frequently used anti-cancer drugs.

## 1. Introduction

Coordinated care of oncological patients plays an important role in the effectiveness of cancer therapy. In recent years, it has been noticed that onconephrological syndromes play a significant role of in the proper diagnosis and therapy of oncological patients who experience nephrological complications in cancer treatment or patients with initial chronic kidney disease qualified for oncological therapy [1]. Nephrologists who consult oncological patients need to possess knowledge of the biology and therapy of individual cancers in order to fully cooperate with the oncologists. Recent years have seen a continuous development of therapy in clinical oncology. A number of new drugs have been introduced for the treatment of cancer, often of very sophisticated structure and action. Dynamic development of oncological therapies causes the improvement of results in the treatment of various cancers as well as longer survival. Unfortunately, the factor which may limit the effectiveness of therapy is the occurrence of various side effects, including nephrotoxicity. The dynamic development of oncological drugs, especially immuno-oncological preparations, results in discovering new pathophysiological mechanisms of potential side effects. The optimal management of a patient requiring oncological therapy should include both the prevention and treatment of possible nephrological complications.

## 2. Materials and Methods

The aim of the publication is analyzing the mechanisms of nephrotoxic effects in oncological therapies, prevention, and treatment of potential complications. For this purpose, the literature in the PubMed, Google Scholar and Medline databases was reviewed using the following keywords: nephrotoxicity, renal failure, acute kidney injury, chemotherapy, and immunotherapy. Special attention has been paid to English-language articles from recent years.

## 3. Chemotherapy

Chemotherapy is a therapy which employs synthetic chemicals (cytostatics) to treat oncological diseases. The employment of this group of substances in oncological treatment results from their impact on the kinetics of neoplastic cells. Depending on the phase of the cell cycle in which the cytostatic operates, they are divided into phase and non-phase dependent [2].

### 3.1. Nephrotoxicity of Chemotherapy—Risk Factors

A number of clinical factors may influence the nephrotoxicity of cytostatic drugs. Significant factors associated with the clinical condition of patients and contributing to nephrotoxicity include both the older age and the female sex. Increased risk in the elderly and women is related to the change in total body water, which decreases when lean body mass is reduced, distorted, elevated estimated glomerular filtration rate (eGFR) despite normal serum creatinine concentration, as well as hypoalbuminemia, which leads to an increase of free drug fractions in blood serum [3]. Due to the high blood flow through the kidneys—approximately 25% of the cardiac output—the kidneys are exposed to high concentrations of drugs. Within the cells of the proximal tubules, a significant drug uptake takes place both through the luminal and basolateral membrane [4,5]. Another important risk factor is hypovolemia, which leads to a reduction in blood flow through the kidneys and leads to hypoxia, which in turn increases the susceptibility of renal tubular cells to damage. The decrease in volemia slows down the flow of the ultrafiltrate through the tubules, which results in higher accumulation of the drug, especially in the renal medulla. Acute renal failure and chronic kidney disease are significant risk factors for impaired renal function in patients with proliferative disease who quality for targeted oncological therapy. The genome of the patient is also important in nephrooncology as it influences allergic readiness to oncological drugs, unfavorable mutations of genes related to metabolism in hepatic and renal enzyme systems (e.g., CYP450) as well as pharmacokinetics favoring the toxicity of the administered drug. Nephrotoxicity is also affected by mutations in the genes of the renal tubular transport proteins [6]. Literature on this topic presents several ways to classify kidney damage caused by various oncology drugs. One of the more commonly used classifications is the division of kidney damage based on the preparation targets in the kidneys. Preparations that damage various structures of the glomeruli in the kidneys, extraglomerular blood vessels and renal tubules have been described [6].

### 3.2. The Assessment of Renal Function in Cancer Patients

The growing population of patients treated with different chemotherapeutic schedules results from the increase in cases of cancer worldwide. The consequence of this fact is an increasing number of undesirable effect cases, including nephrotoxicity. Therefore, the reliable methods of kidney function monitoring seems to be an extremely important element of oncology care [7]. The serum creatinine level is still a key parameter of renal function assessment. The criteria of acute kidney injury (AKI), acute kidney disease (AKD) as well as chronic kidney disease (CKD), including newly proposed definition of AKI—are based on creatinine concentration [8]. The cystatin C whose serum level depends on GFR is the alternative substance that may be used for kidney function monitoring. However, it was reported that cystatin C measurements may be affected by a number of causes—such as anthropometric data, thyroid disease, diabetes or corticosteroids [9]. Moreover, the independent effects of both malignancy and chemotherapy on cystatin C levels were revealed. This indicates that cystatin C alone is not an appropriate marker of kidney function. The estimation of GFR in clinical practice is usually performed using regression equations. The available formulas include: Cocroft-Gault, MDRD (Modification of Diet in Renal Disease), CKD –EPI creatinine (Chronic Kidney Disease Epidemiology Collaboration), CKD-EPI cystatin C, CKD-EPI creatinice-cystatine C. The cancer-specific GFR equations—such as Martin formula and Calvert formula were also developed. However, these formulas are not widely accepted for clinical use. The studies comparing CKD-EPI equation with Cocroft-Gault and MDRD equations revealed that CKD-EPI is more precise than other formulas (especially Cocroft-Gault) in the assessment of GFR [10,11].

The CKD-EPI creatinine formula is as follows:

eGFR = 141 × min(S_Cr_/κ, 1)^α^ × max(S_Cr_/κ, 1)^−1.209^ × 0.993^Age^ × 1.018 [if female] × 1.159 [if Black]

where:

κ = 0.7 (females) or 0.9 (males)

α = −0.329 (females) or −0.411 (males)

min = indicates the minimum of S_Cr_/κ or 1

max = indicates the maximum of S_Cr_/κ or 1

age = years.

The CKD-EPI creatinine-cystatine formula may be used in some cases when the assessment error should be minimized. The formula is as follows:

eGFR = 135 × min(S_Cr_/κ, 1)^α^ × max(S_Cr_/κ, 1)^−0.601^ × min(S_cys_/0.8, 1)^−0.375^ × max(S_cys_/0.8, 1)^−0.711^ × 0.995^Age^ × 0.969 [if female] × 1.08 [if black]

where:

eGFR (estimated glomerular filtration rate) = mL/min/1.73 m^2^

S_Cr_ (serum creatinine) = mg/dL

S_cys_ (standardized serum cystatin C) = mg/L

κ = 0.7 (females) or 0.9 (males)

α = −0.248 (females) or −0.207 (males)

min(S_Cr_/κ or 1) = indicates the minimum of S_Cr_/κ or 1

max(S_Cr_/κ or 1) = indicates the maximum of S_Cr_/κ or 1

min(S_cys_/0.8, 1) = indicates the minimum of S_cys_/0.8, 1

max(S_cys_/0.8, 1) = indicates the maximum of S_cys_/0.8, 1

age = years.

The real-time GFR assessment may provide more accurate monitoring of kidney function [12]. There are two methodologies in development that allow for such assessment. The first of them uses a new biomarker that consists of 150-kD rhodamine derivative and smaller 5-kD fluorescein carboxymethylated dextran [13]. The plasma concentrations of both compounds are measured at the given time points using a fluorimeter. The second method is a transdermal GFR measurement system which also uses the fluorescence [14]. These methods may contribute to more effective monitoring of kidney function and better personalization of therapy in the near future.

### 3.3. Chemotherapeutics

The most commonly administered chemotherapeutic agents that can damage the kidneys are listed below. An attempt was made to present the pathomechanism of nephrotoxic effects of selected oncolological drugs, clinical symptoms accompanying kidney damage, as well as prevention and treatment options. The most commonly used conventional agents include alkylating agents, cytotoxic antibiotics and antimetabolites.

#### 3.3.1. Group of Alkylating Agents

##### Platinum-Based Chemotherapeutic Drugs

Cisplatin is a widely used chemotherapeutic agent in oncology. The mechanism of its action is based on the inhibition of DNA synthesis by creating bonds preventing the replication of the DNA chain [15]. It is used in monotherapy and complex treatment of malignant neoplasms such as lung, testicular, bladder, head and neck cancer in palliative and radical intent of treatment [16].

One of the main factors limiting its use is its nephrotoxicity, which depends on the cumulative dose of cisplatin taken [17]. The other risk factors which increase the risk of nephrotoxicity include dose and frequency of the drug administration, older age, female sex, smoking, hypoalbuminemia, pre-existing renal insufficiency. The risk factors associated with lower risk include diabetes (uncertain in humans) and OCT2 polymorphism [17]. The development of nephrotoxicity occurs most often in the 2nd week of use and affects approximately 30% of patients [18]. Nephrotoxicity of the drug is clinically manifested as: acute kidney damage, development or worsening of chronic kidney disease, hypomagnesemia, hypokalemia, hypocalcemia or renal salt-wasting syndrome [17,19]. Special attention needs to be paid to electrolyte disturbances, which should be particularly considered when administering high doses of cisplatin [19]. The most common electrolyte disorder is hypomagnesemia caused by magnesium malabsorption in the ascending limb of the loop of Henle and the distal tubule [20].

The mechanism of kidney damage and cisplatin-induced electrolyte disturbances is complex and not fully known. The role of the organic cationic transporter type 2 (OCT-2) located on the surface of renal tubular cells is postulated in the development of hypomagnesemia. As a result of its activity, a significant accumulation of cisplatin and its metabolites occurs, which leads to damage to the tubular cells [21]. Additionally, two other processes are taken into account in the process of kidney damage: damage to small renal vessels (which causes ischemia of the renal parenchyma) and induction of inflammation [18].

Carboplatin is a second-generation drug, with much lower nephrotoxicity than cisplatin, which results from the different structure of the drug molecule (the presence of a carboxyl group instead of chlorine). It is associated with a lack of affinity with OCT-2 and reduced accumulation in renal tubular cells [22]. Therefore, in palliative intent of treatment may be used as a substitute for cisplatin as it causes less frequent occurrence of nephrotoxicity and electrolyte disorders [23].

The clinical significance of hypomagnesemia is not fully understood as its symptoms may be masked by the underlying disease. Some authors point to the beneficial effect of magnesium supplementation in fluid therapy employed before and after the administration of cisplatin/carboplatin (magnesium supplementation reportedly reduces the toxic effect of these drugs on the kidneys) [20,24].

In the prevention of nephrotoxicity of cisplatin and carboplatin, the most important factors are correct fluid therapy, correction of accompanying electrolyte disturbances with careful monitoring of renal function and diuresis, and adjustment of the drug dose to renal function.

Oxaliplatin is commonly used compound in adjuvant and palliative chemotherapy regimens in colorectal cancer. In contrast to other platin compounds the nephrotoxicity occurs rarely (<5%, <1% severe). Oxaliplatin may cause renal tubular vacuolization, acute tubular necrosis, renal tubular acidosis, and acute kidney injury [25,26,27]. The incidence of adverse event during oxaliplatin based chemotherapy correlates with renal function [28].

##### Nitrogen Mustards

Cyclophosphamide is a cytostatic agent from the group of oxazophosphites with an alkylating effect. It is used both in monotherapy and in complex therapeutic regimens, such as treatment of leukemias, lymphomas or solid tumors (breast cancer), as well as in some autoimmune diseases [29].

One of the most serious side effects of using cyclophosphamide is the risk of causing hemorrhagic cystitis [30]. The mechanism of this complication is associated with the liver metabolism of cyclophosphamide (which is a prodrug), resulting in the formation of active metabolites: phosphoramide mustard and the toxic acrolein. The latter substance is filtered in the kidneys and accumulates in the bladder. Acrolein causes cell death by inducing inflammation (increases the production of reactive oxygen species and nitric oxide by increasing the activity of nitric oxide synthase in the cells, which results in forming peroxynitrates damaging the proteins and lipids) and leading to the development of hemorrhagic cystitis [31]. Cyclophosphamide is relatively safe for the kidneys and is administered, inter alia, in the steroid-dependent nephrotic syndrome in children [32].

The literature also describes rare cases resembling the syndrome of inadequate antidiuretic hormone secretion (SIADH) caused by the use of cyclophosphamide (related to hyponatraemia, in the course of an increase in the amount of free water in the body). It is postulated that cyclophosphamide stimulates the secretion of antidiuretic hormone (ADH), which results in impairing free water secretion through renal tubules [33].

Ifosfamide is a cytostatic agent, prodrug, and cyclophosphamide analogue. It is administered both in monotherapy and complex cancer therapeutic regimens, among others. in an advanced stage of testicular cancers and sarcoma.

Nephrotoxicity in the course of using this medicine is well known and may manifest itself as: acute kidney damage, irreversible intensification of chronic kidney disease, hemorrhagic cystitis or dysfunction/damage of proximal renal tubules [34]. The last complication manifests clinically with hypokalemia, hypophosphatemia, hypouricemia, glycosuria, metabolic alkalosis and tubular proteinuria. Some of these changes are reversible [34]. Ifosfamide is transported by organic cationic transporter (OCT-2) located on the surface of renal tubular cells to their interior [5]. As a result of its accumulation in the renal tubular cells, its metabolism takes place and the formation of two nephrotoxic metabolites of acrolein and chloracetalaldehyde occurs, which leads to damage to the renal tubular cells and (as in the case of cyclophosphamide) hemorrhagic cystitis [35].

In order to reduce nephrotoxicity, the doses of cyclophosphamide and ifosfamide are adjusted to the degree of renal function. Mesna infusions and adequate hydration of the patient to stimulate diuresis are used in the prophylaxis of hemorrhagic cystitis [36]. Mesna (2-mercaptoethanesulphonic acid sodium salt) is a substance with a mucolytic effect and at the same time an antidote to the harmful effects of acrolein. It combines with acrolein, resulting in a new compound that becomes non-toxic and easily soluble in water, which facilitates its excretion from the body [37]. According to the recommendations of the manufacturer, when using ifosfamide, mesna should always be used, and in the case of cyclophosphamide, if the administered dose of the drug exceeds >10 mg/kg of body weight.

##### Nitrosourea Derivatives

Nitrosourea derivatives belong to a large group of alkylating drugs and includes lomustine, carmustine and prokarbazine. The mechanism of action is based on the formation of a highly reactive alkyl-carbonate cation covalently bonding with electrophilic functional groups such as amino, carboxyl, phosphate and thiol groups, proteins and purines that build deoxyribonucleic acid (DNA). This reaction causes the alkylation of the nitrogen atoms of guanine N7 and N1, adenine N1 and N3 as well as cytosine N3. This leads to numerous changes in the structure of the genetic material resulting in abnormal base pairing by incorrectly combining guanine with thymine, loss of guanine or adenine from the DNA strand (depurination) and cleavage of the DNA strand. Changes in the structure of DNA inhibit or prevent DNA transcription, synthesis, and block cell division [38].

Kidney damage caused by drugs from this group is not very common and depends mainly on accompanying factors such as gender (females), age over 65, presence of nephrotic syndrome, hypoalbuminemia, mechanical jaundice, history of kidney disease, decreased glomerular filtration (e.g., dehydration), metabolic disorders causing changes in urine pH, multi-drug chemotherapy, polymorphism of the CYP450 system (hepatic and renal). Nephrotoxicity in the case of nitrosourea derivatives is chronic and becomes apparent after a certain period of time since the administration of the therapy [23,39]. The pathogenesis of nephrotoxicity includes the appearance of active oxygen radicals and an increase in metabolism (high metabolism) of the renal tubular cells. At the same time, an uptake of toxic metabolites of chemotherapeutic agents occurs in the proximal tubule through endocytosis and pinocytosis as well as the basolateral transport of organic anionic and cationic compounds. Nephrotoxicity increases long-term exposure to toxic substances associated with the periodicity and duration of chemotherapy administration (multiple cycles of chemotherapy). In the prevention of toxicity of nitrosourea derivatives, the current state of kidney function should be taken into account, and the medical history, especially in terms of kidney diseases, should be carefully analyzed in order to reduce the possibility of drug interactions related to nephrotoxicity. Each patient should be monitored clinically, particularly for adequate hydration, and normalization of possible protein and metabolic disorders (urine pH should be alkaline). A very important aspect is the monitoring and appropriate adjustment of clinical and laboratory parameters that may affect the occurrence of renal complications.

#### 3.3.2. Cytotoxic Antibiotics

Mitomycin C is an antibiotic produced by Streptomyces caespitosus. Its mechanism of action is based on alkylation and inhibition of DNA synthesis [40]. In clinical practice it is used in anal cancer treatment.

The nephrotoxicity of mitomycin C is well known [41]. During its use, acute kidney damage, irreversible deterioration of kidney function (GFR decrease), proteinuria, hematuria and the most serious complication which is thrombotic thrombocytopenic purpura (TTP)/hemolytic uremic syndrome (HUS) may occur [42]. Spontaneous HUS may occur in the course of many advanced neoplastic diseases, among the drugs used in chemotherapy it is most frequently caused by mitomycin [43]. On the other hand, the risk of developing HUS depends on the cumulative dose of the drug taken. The toxicity of mitomycin results from its direct damaging effect on vascular endothelial cells [44]. The development of this complication takes place most often within a few months of drug administration.

In order to counteract the occurrence of mytomycin-induced HUS, the authors of some studies recommend not to exceed the cumulative dose of the drug > 60 mg/m^2^ and to maintain an interval of several weeks between successive doses [45]. The use of plasmapheresis and steroid therapy in the treatment of the above complication (efficacy not confirmed) allows for the inhibition of thrombotic microangiopathy, but does not affect the recovery/improvement of kidney function.

#### 3.3.3. Antimetabolites

##### Methotrexate (4-amino-4-deoxy-N10-methylfolic acid)

Methotrexate (4-amino-4-deoxy-N10-methylfolic acid) is a chemical compound that is an antagonist and a structural analog of folic acid. The anticancer mechanism of methotrexate is based on high affinity for dihydrofolate reductase and blocking its action. The active compound is a methotrexate metabolite, which is 7-hydroxymethotrexate (7-OH-MTX) and 4-amino-4-deoxy-N10-methylopteran acid (DAMPA) as well as polyglutamine derivatives, which increase the potency of the drug. As a result, the synthesis of nucleic acids (DNA and RNA) and proteins is disturbed in the cell, which leads to cell death. The cytotoxic effect is cell cycle dependent and is active only in the S phase of the cell cycle. Only in the case of high doses the drug can act on the cell in the G1 phase of the cycle. The cytotoxic effect depends on the time of exposure to the drug, therefore most chemotherapy regimens consist of an hours-long intravenous infusion of the drug [46]. In the case of methotrexate, the mechanism of nephrotoxicity is related to the precipitation (crystallization) of the methotrexate metabolite (mainly 7-OH-MTX) in the renal tubules. Additionally, disturbances in the vascular resistance of the supplying glomerular arteries (reduced perfusion) and direct damage to the glomeruli and renal tubules are assumed as factors increasing the nephrotoxicity of methotrexate. Methotrexate is actively filtered and secreted by the renal tubular cells [47,48]. Thus, prior renal dysfunction is a serious risk factor for nephrotoxic complications. The pathophysiology of nephrotoxicity takes into account the increase in the activity of myeloperoxidase, antioxidant enzymes such as superoxide dismutase, glutathione peroxidase and malondialdehydes, which leads to cell damage caused by free oxygen radicals. At the same time, an increased response from neutrophils (tissue infiltrations) occurs, which ultimately leads to kidney damage [47,49]. Due to the development of molecular biology, searches for mutations in genes responsible for the occurrence of nephrotoxicity have been recently conducted. The main lines of research concern the polymorphism of genes related to proteins responsible for transport to and from cells, polyglutamination of methotrexate and folate metabolism. The risk factors for nephrotoxicity in the case of the administering methotrexate include: hydration status, previous kidney diseases (kidney damage), metabolic disorders (acidosis), drugs used and their interactions with methotrexate (NSAIDs, procarbazine, penicillin derivatives, sulfonamides). Clinical symptoms of methotrexate-associated nephrotoxicity (1.8% of patients treated with high doses) are: in the first phase, non-oliguric renal dysfunction, in most cases reversible, manifested by decreased glomerular filtration rate, albuminuria and increased concentrations of tubular enzymes (NAG, AAP) [50]. To avoid nephrotoxicity, proper hydration (2.5–3.0 L of fluids daily 12 h before the start of therapy) and urine alkalinization (pH > 7.5) should be introduced, and drugs that increase nephrotoxicity should be eliminated. The second basic treatment is the use of leucovorin (folinic acid). Leucovorin is a racemic mixture of N^5^-formyl-THF stereoisomers which, when administered intravenously, is converted to the major active folate of the human N^5^-methyl-THF. This compound prevents the inhibition of thymidine synthesis, and thus DNA synthesis. Leucovorin is administered 24 h after the end of the methotrexate infusion over a period of 48 h. In all cases, serum methotrexate levels should be assessed in order to modify the leucovorin dose (24, 48 and 72 h after the end of the infusion). At the same time, urine pH and laboratory parameters should be monitored. The safe concentration of methotrexate is 0.05 μM. This type of procedure prevents the occurrence of renal complications. In the case of complications in the treatment, it is recommended to use high doses of leucovorin, carboxypeptidase G_2_ (CPDG_2_) and thymidine as well as dialysis therapy (limited efficacy); Late nephrotoxicity may occur when methotrexate is administered. This type of complication is associated with the cumulative dose of metrexate, defined as between 16–45 g/m^2^. The main complication of late nephrotoxicity is decreased glomerular filtration rate and albuminuria [51,52].

##### Pemetrexed

Pemetrexed is a drug that is active as a folic acid antagonist. It works by inhibiting the proper synthesis of nucleic acids (DNA and RNA). It acts by inhibiting the enzymes essential for the synthesis of purine amines: thymidylate synthetase (TS), dihydrofolate reductase (DHFR), and glycinamide ribonucleotide formyltransferase (GARFT). Due to inhibiting the action of these enzymes, an incorrect synthesis of purine amines, thymidine and pyrimidine occurs, and thus disturbances in cell growth and induction of apoptosis. This process takes place both in normal (healthy) and cancer cells [53]. This drug has found application in the treatment of lung (NSCLC) and pleura (mesothelioma) cancers [53,54]. The nephrotoxic effect has its pathomechanism in inducing stromal fibrosis, interstitial edema, and vacuolization of proximal tubular cells with the retained glomerular structure [13,55,56]. As a late complication, thinning of the cortical layer and a decrease in the corticomedullary are observed. The pathomechanism of this type of changes has not been fully explained. It is assumed that the disturbances of PEM (folate receptor α) uptake, reduced folate carrier (RFC) in proximal tubular cells is responsible for the occurrence of nephrotoxicity in the case of the use of pemetrexed. Additional causes of nephrotoxicity include decreased glomerular filtration rate in the course of muscle mass loss, dehydration, hypoalbuminemia and interactions with potentially nephrotoxic drugs [56,57,58,59]. Histopathological examinations of patients with features of kidney damage show renal tubular necrosis and atrophy as well as interstitial changes (from inflammation to fibrosis). Laboratory tests demonstrate proteinuria, glycosuria, leukocyturia corresponding to diabetes insipidus and distal tubular acidosis. There are no clear recommendations for the treatment of complications. A minority of patients require hemodialysis, some authors advise administration of thymidine in severe cases. A very important aspect of preventing nephrotoxicity is taking into account the loss of muscle mass and its effect on glomerular filtration (eGFR) as well as the correction of metabolic disorders before starting therapy [55,58,60].

##### Gemcitabine

Gemcitabine is a cytostatic agent belonging to the group of antimetabolites (a pyrimidine nucleoside analog) It is administered in the treatment many different cancers including urothelial bladder cancer, pancreatic adenocarcinoma, ovarian cancer or non-small cell lung cancer [61,62,63,64].

Chronic irreversible renal failure due to hemolytic uremic syndrome (HUS) may develop during treatment with gemcitabine [65]. HUS includes hemolytic anemia, thrombocytopenia and acute kidney injury [66]. The mechanism of developing this complication during gemcitabine use is unknown. Treatment of HUS is based, among others, on steroid therapy and plasmapheresis, but the applied treatments frequently do not lead to an improvement in kidney function [67]. Regular control of renal function is recommended during treatment with gemcitabine.

##### 5-Fluorouracil

5-fluorouracil is a nucleobase analogue in which at the 5-position/position 5 hydrogen has been replaced by fluorine. Due to the change in chemical structure, this compound inhibits DNA synthesis by blocking the conversion of deoxyuridyl acid to thymidylic acid via thymidylate synthetase. It is an antimetabolite to pyrimidine. 5-fluorourocil itself and its metabolites exhibit antitumor activity. 5-fluorooxy uridine monophosphate (F-UMP) inhibits RNA processing, and 5-5-fluoro-2′-deoxyuridine-5′0-monophosphate (F-dUMP) inhibits thymidylate synthetase, which causes depletion of thymidine triphosphate (TTP), which results in inhibiting DNA synthesis. At the same time, it is a chemical compound that sensitizes cells to the effects of ionizing radiation, administered immunosuppressive and anticancer agents [68,69]. In the conducted studies on the pathogenesis of nephrotoxicity, the relationship between the expression of miR-181 and the nephrotoxicity of 5-flurouoracil was confirmed. Over-expression of this gene in mesangial cells during treatment with 5-fluorouracil is associated with a decrease in the inhibition of the BIRC6 protein and thus an increase in cell apoptosis. This mechanism is conditioned by an increase in p53-associated mitochondrial apoptosis (including decreased Bcl-2/Bax ratio, loss of mitochondrial membrane potential, cytochrome C release and activation of the caspase system (caspase 6 and 3) [68,70]. The nephrotoxicity of 5-fluorouracil causes disturbances in the hemodynamics of the intraglomerular circulation, damage and necrosis of renal tubular cells, nephropathy due to crystallization of chemotherapeutic agents, kidney damage resulting from rhabdomyolisis and thrombotic thrombocytopenic purpura (TTP/HUS). Histopathological examinations reveal microbleeding and vacuolization of the glomerular cells. Laboratory tests show increased creatinine levels with normal urea concentration. Carvedilol, prednisone, diltiazem and L-arginine can be used to prevent nephrotoxicity. In prevention, water-electrolyte and metabolic disturbances should be corrected [70,71].

## 4. Immunotherapy as a New Method of Cancer Treatment

Advances in basic science led to improvements in our knowledge about the mechanisms of cancer growth and development. Avoiding immune destruction by altered immune recognition has been recently described as a classical hallmark of cancer [72]. As a result, we can better understand the mechanisms of cancer progression and improve the possibilities of treatment. Immunotherapy has become a new tool for cancer therapy, significantly differing from classical chemotherapeutics. Immune therapy is an example of targeted therapy and is used in several cancers like lung, kidney, bladder, head and neck cancers, as well as melanoma (Table 1). TNFα and IL-2 was the first immunotherapy that was registered for treating renal cell carcinoma or melanoma. However, its efficacy was moderate and toxic [73]. Nowadays immune checkpoint inhibitors (ICPs) are the mainstay of immunotherapy.

Programmed death receptor 1 (PD-1) and cytotoxic T-lymphocyte-associated protein 4 (CTLA-4) are molecules which regulate immune response and prevent overactivation and development of autoimmunity. CTLA-4 is expressed only on T cells and regulates the early immune response. PD-1 receptor is present on the surface of antigen-presenting cells (APC), T cells, B cells, NK, monocytes, dendritic cells and regulates later immune response. The presence of PD-L1 on tumor cells decreases the activity of T- lymphocyte. Pharmacological inhibition of CTLA-4 and disruption of the PD-1/PD-L1 axis enhance the anti-tumoral activity of lymphocytes [74].

Anti-PD1 (nivolumab, pembrolizumab), anti-PDL1 (durwalumab, atezolizumab, avelumab), and anty-CTLA4 (ipilimumab) antibodies are used in radical and palliative cancer treatment in several indications. Many clinical trials confirmed clinical benefits for patients treated with ICPs inhibitors. The side effects of immunotherapy differ from side effects of chemotherapy. They are unpredictable and do not depend on the cell cycle. Classically immune-related side effects can be divided into infusion reaction and immune-related adverse events (irAE). They include skin toxicity, endocrinopathies, hepatotoxicity, gastrotoxicity, pneumonitis, which occur more frequently than neurological, cardiac, rheumatological, hematological, ocular, and renal toxicity [75].

### 4.1. Nephrotoxicity of Immunotherapy

The clinical problems related to kidney injury can be divided into volume depletion, acute and chronic kidney injury, electrolyte disorders, microangiopathies, nephritic syndrome, tumor lysis syndrome toxicity of chemotherapeutics and non-chemotherapeutics [76]. Among several targeted therapies directed against EGFR, HER2, PD1/PDL1 and CTLA-4 the most nephrotoxic agents are ipilimumab and cetuximab [77].

Nephrotoxicity is one of the classical side effects of cisplatin, which is used as an alkylating agent in several cancers like lungs cancer, bladder cancer, head and neck cancers [78]. Nephrotoxicity is a major factor, limiting the use of cisplatin in cancer treatment, which classically requires eGFR >60 according to the criteria of eligibility for cisplatin. There are several differences between cisplatin toxicity and immunotherapy. The mechanism of kidney injury after cisplatin treatment involves tubular death due to inflammation, vascular injury and activation of death promoting signaling pathways. The other differences include biochemical symptoms (decrease of glomerular filtration rate and magnesium, potassium disorders), risk factors and method of prevention (hydration) [18].

### 4.2. Types and Epidemiology of Immune-Related Nephrotoxicity

Several renal and urinary disorders were reported in clinical trials with ICPs inhibitors. They included AKI, autoimmune nephritis, dysuria, nephrolithiasis, toxic nephropathy, prerenal failure, renal colic, renal failure, renal impairments, tubulointerstitial nephritis, urinary retention, hematuria, renal tubular nephritis and glomerulonephritis [79]. In contrast to interstitial nephritis, glomerulopathies with different histological pattern occur much less often. However, some case report studies were published presenting membranoproliferative glomerulonephritis, minimal change disease, pauci-immune glomerulonephritis, membranous glomerulonephritis, C3 glomerulonephritis, immunoglobulin A nephropathy, amyloid A amyloidosis, focal segmental glomerulonephritis and lupus nephropathy [80,81,82].

The occurrence of nephrotoxicity upon ICPs inhibitors varied between 1 to 22% and occurred more frequently in anti-PD1 than anti-CTLA-4 therapy. Among irAEs nephrotoxicity is one of the rarest and reaches < 1% of patients treated with ICPs inhibitors, but, the incidence may be underestimated. Recent analysis of 48 clinical trials and 11,482 patients treated with anty- PD-1 (nivolumab and pembrolizumab) shows, that incidence rate of acute kidney injury is 2.2% [83]. In Oleas and al. review of 826 patients treated with immunotherapy, 15.1% developed AKI and among them 18.5% needed hospitalization [84]. Due to distinction in the study group, type of cancer, and differences between accessed outcomes direct comparison of kidney immune-related adverse events is difficult to manage. The incidence of immune-related kidney injury depends on treatment regimen (monotherapy or polytherapy) and type of medicine (anti -PD-1, anti-PD-L1, anti -CTLA4). In monotherapy, kidney injury occurs more rarely than polytherapy. Nephrotoxicity occurred most frequently in the Nivolumab-Ipilimumab combination and reached 4.9%, but was mostly graded 1 or 2 according to CTCAE criteria [75]. Importantly, the connection of cisplatin-based chemotherapy regimen with anti-PD-L1 therapy did not alter significantly ototoxicity and nephrotoxicity except a mild worsening of distortion product otoacoustic emissions and a mild increase in serum creatinine [85]. Time to a development of kidney injury is different and may occur just after a single dose of ICPi, but varies between studies from 3 weeks to 8 months. Nephrotoxicity may be isolated or be one of the several irAE. Secondary irAE usually occurs prior to AKI but may occur at the time or after AKI [81]. In the case series study the hypothyroidism was the most frequent synchronic irAE.

### 4.3. Mechanism of Nephrotoxicity

Nephrotoxicity during immunotherapy may be CTLA-4 and PD1 related but contrary to renal cell carcinoma, kidney cells have only low expression of PD-L1 in proximal segments of tubules [86]. Studies on the animal model show that mice lacking CTLA-4 developed lethal lymphoproliferative disorders [87]. Animals depleted of PD1 spontaneously developed lupus-like proliferative arthritis and glomerulonephritis with predominant IgG3 deposition [20]. The mechanism of kidney injury following ICP inhibitors is mostly based on tubule-intestinal damages by cytotoxic cells. Nephrotoxicity as a result of PD-L1 inhibition may be the result of reactivation or formation of T cells, which led to T cell proliferation, cytotoxicity, cytokine releasing, complement-mediated inflammation and potentially autoimmune reaction [82]. The precise mechanisms of autoimmune side effects of immunotherapy remain unknown but may depend on polymorphisms of the molecules [83]. Characteristics of inflammatory kidney infiltration may be precisely described with immunohistochemical studies or flow cytometry. Biopsy studies show that acute tubulointerstitial nephritis induced by ICP inhibitors consists of mononuclear cell infiltration (CD3 + T cells, CD4 + T helper cells, CD68 + macrophages, CD163 + macrophages), plasma cells, eosinophils, vasculitis lesions, or sometimes deposits of immunoglobulins and complements. Non-specific granulomatous features were also described as well. Infiltration of CD8+ cytotoxic T cells, CD20+ B cells, and CD1c+ dendritic cells were less expressed [88]. The late stage of tubulointerstitial injury is fibrosis. Additionally, kidney biopsy flow cytometry may be performed to better characterize the immune response in the blood. Analysis of patients who developed kidney injury after nivolumab and ipilimumab showed highly proliferative CD4+ and CD8+ lymphocytes. Moreover, levels of T memory cells were also increased [89]. There are reports which suggest that proton pomp inhibitors therapy may predispose to AKI. ICPs may induce drug specific T cell, which recognizes drug antigen and leads to kidney injury [90].

### 4.4. Diagnosis of Nephrotoxicity

The clinical symptoms of immunotherapy driven AKI are not specific. Diagnosis of kidney injury is based on laboratory tests (creatine, urea, gasometry- metabolic acidosis, electrolytes) and measurement of urine output. Additional laboratory tests include morphology (eosinophilia) and urinalyses (infection, proteinuria, hematuria, pyuria, hematuria). The most common alteration is sterile pyuria and sub-nephrotic proteinuria. Patients with grade 2 AKI may be tested for glomerulonephritis. The anti-nuclear antibodies, complement C3, C4, anti-neutrophil cytoplasmic antibodies, anti-glomerular basement membrane, hepatitis, HIV, immunoglobulins, and protein electrophoresis should be considered. USG and USG doppler should also be considered to exclude obstruction and clotting. Differential diagnosis of AKI includes prerenal (hypovolemia (diuretics, infection, diarrhea), cardiotoxicity (cardiomyopathy, heart failure, pulmonary hypertension, hypo and hypertension)), renal (drugs (CT contrast, proton-pump inhibitors, nonsteroidal anti-inflammatory drugs, antibiotics) and postrenal kidney injury (obstructive (stones, benign prostatic hyperplasia). Kidney biopsy should be considered to exclude other reasons of AKI than irAE to determine a need for steroids, because administration of high dose steroids in patients treated with immunotherapy may decrease effects of treatment [84]. Biopsy is recommended since grade 2 toxicity. The material should be stained with haematoxilin and eosin, periodic acid–Schiff and Masson’s trichrome. Cases confirmed by biopsy show that the most common type of nephrotoxicity is interstitial nephritis with damage to tubules and less frequently glomerulopathies. The most common histopathological findings were edema, interstitial inflammation and tubulitis [88]. The ICPI-associated AKI may be defined according to Common Terminology Criteria for Adverse Events (CTCAE), which depends on changes in creatinine according to upper limit of normal or baseline. AKI has 4 levels (grade 1 to 4). Grade 1 irAE means that creatinine level increases between upper limit of normal (ULN) to 1.5 times above ULN. Grade 2 AKI is defined as creatinine elevation 1.5–3 times above the baseline or ULN. In grade 3 AKI, creatine is 3 times above the baseline or 3–6 times above ULN. The life-threatening grade 4 kidney injury occurs when creatinine is 6 times above the ULN. Hospitalization is recommended for patients with grade 3 toxicity.

### 4.5. Treatment

Steroids are basic drugs used in treatment of irAE. Prednisone (0.5–1 mg/kg/day) and methyl prednisone (1–2 mg/kg/day) are recommended in treatment of ICPs-related toxicity, but in some circumstances hydrocortisone also was used. According to case reports, treatment with steroids treatment often led to partial or complete remissions of disease, but relapses were also reported. Remission of disease is defined as an improvement of creatinine level (complete recovery- creatinine less than 0.35 mg/dL above the baseline, partial recovery- creatinine between baseline + 0.35 mg/dL and less than 2 times of baseline) [81]. Corticosteroids reduce the risk of kidney fibrosis, which may be related to chronic renal failure. Due to the risk of adrenal insufficiency, steroid withdrawal should be gradual. If steroids have been taken due to grade 2 toxicity, then steroid withdrawal should last over 2–4 weeks. In case of grade 3 and 4 toxicity, steroid withdrawal should last over 4 weeks. In case of insufficiency of steroids, other drugs should be considered to manage the toxicity such as azathioprine, cyclophosphamide, cyclosporine, infliximab and mycophenolate (NCCN). Plasmapheresis may be performed as well.

## 5. Conclusions

The increased number of patients receiving oncological treatment results from the growing cancer incidence which is observed worldwide throughout recent years. Chemotherapy as well as immunotherapy are key elements of oncology care. Unfortunately, these therapies lead to different complications in some patients, including nephrotoxicity. Immunotherapy is a milestone in modern oncology. Its undesirable effects are different than in classical chemotherapy. However, some immune-related adverse events (irAE) may be life-threatening. The validation of different combinations of immune- and chemotherapy is a subject of numerous ongoing clinical trials. The growing role and applications of immunotherapy is expected in the next future and that is why the incidence of irAE may be increased.

Although renal failure does not belong to leading complications of oncotherapy, its clinical importance is very high because it may be a reason of temporary or permanent discontinuation of treatment which affects clinical outcome. Therefore, both oncologists and nephrologists should be well educated and experienced in management of nephrotoxic effects, including:

adequate monitoring of kidney function in cancer patients
application of available preventive measures reducing the risk of kidney damage
appropriate treatment of renal complications.

Finally, it should be underlined that despite the progress in diagnostics and treatment of kidney failure, especially AKI, many problems are still unsolved and many questions remain unanswered.

## Figures and Tables

**Table 1:** Indications and mechanism of action ICPs.

Name of Drug	Mechanism of Action	Indication
Ipilimumab	Anti CTLA-4	Melanoma RCC Mesothelioma Renal cell carcinoma Colorectal cancer MSI-H HCC NSCLC Esophageal cancer
Nivolumab	Anti PD1	Melanoma RCC NSCLC and SCLC Head and neck cancer Hodgkin lymphoma HCC Colorectal cancer MSI-H Gastric, Gastroesophageal junction and esophageal cancers Mesothelioma Urothelial carcinoma
Pembrolizumab	Anti PD1	Melanoma NSCLC and SCLC Head and neck cancer Hodgkin lymphoma Primary Mediastinal Large B-Cell Lymphoma Urothelial Carcinoma MSI-H Cancer Gastric and Esophageal Cancer Cervical and Endometrial Cancer HCC Merkel cell carcinoma Breast cancer Cutaneous skin cancer MSI-H cancer RCC
Avelumab	Anti PD1	Merkel cell carcinoma Urothelial carcinoma RCC
Atezolizumab	Anti PD-L1	Urothelial Carcinoma NSCLC and SCLC Melanoma HCC
Durvalumab	Anti PD-L1	Urothelial Carcinoma NSCLC Biliary track cancer

Abbreviations: HCC: hepatocellular cancer, MSI-H- microsatellite instability high, NSCLC/SCLC: non small/small cell lung cancer, RCC- renal cell carcinoma, TNBC- triple negative breast cancer.
